# Performance of a System for Rapid Phenotypic Antimicrobial Susceptibility Testing of Gram-Negative Bacteria Directly from Positive Blood Culture Bottles

**DOI:** 10.1128/jcm.01525-22

**Published:** 2023-02-28

**Authors:** J. Göransson, M. Sundqvist, E. Ghaderi, J. G. Lisby, Y. Molin, E. Eriksson, S. Carlsson, A. Cederlöf, L. Ellis, J. Melin

**Affiliations:** a Q-linea AB, Uppsala, Sweden; b Department of Laboratory Medicine, Clinical Microbiology, Faculty of Medicine and Health, Örebro University, Örebro, Sweden; c Department of Bacteriology, Uppsala University Hospital, Uppsala, Sweden; d Department of Clinical Microbiology, University of Copenhagen, Hvidovre Hospital, Hvidovre, Denmark; NorthShore University HealthSystem

**Keywords:** antimicrobial susceptibility testing, MIC, phenotypic AST, rapid AST

## Abstract

The rapid administration of optimal antimicrobial treatment is paramount for the treatment of bloodstream infections (BSIs), and rapid antimicrobial susceptibility testing (AST) results are essential. Q-linea has developed the ASTar system, a rapid phenotypic AST device. Here, we report the performance of the ASTar BC G− (Gram-negative) kit when assessed according to the ISO 20776-2:2007 standard for performance evaluation of *in vitro* diagnostic AST devices. The evaluated ASTar BC G− kit uses a broad panel of 23 antimicrobials for the treatment of BSIs caused by Gram-negative fastidious and nonfastidious bacteria across a range of 6 to 14 2-fold dilutions, including cefoxitin as a screening agent for AmpC-producing *Enterobacterales*. The ASTar system processes blood culture samples to generate data on MICs and susceptible, intermediate, or resistant (SIR) category. The automated protocol includes concentration determination and concentration adjustment to enable a controlled inoculum, followed by broth microdilution (BMD) and microscopy performed continuously to generate MIC values within approximately 6 h once the test is run on the ASTar system. The performance of the ASTar system was assessed against the ISO 20776-2:2007 standard BMD reference method. Testing was performed across three sites, with results from 412 contrived blood cultures and 74 fresh clinical blood cultures. The ASTar system was also tested for reproducibility, with triplicate testing of 11 strains. The accuracy study comprised 8,650 data points of bacterium-antimicrobial tests. The ASTar system demonstrated an overall essential agreement (EA) of 95.8% (8,283/8,650) and a categorical agreement (CA) of 97.6% (8,433/8,639) compared to the reference BMD method. The overall rate of major discrepancies (MDs) was 0.9% (62/6,845), and that of very major discrepancies (VMDs) was 2.4% (30/1,239). This study shows that the ASTar system delivers reproducible results with overall EA and CA of >95%.

## INTRODUCTION

Bloodstream infection (BSI) represents a significant global burden of disease, with an estimated 157,000 deaths annually in Europe alone (1). BSI can lead to sepsis, a life-threatening organ dysfunction due to a dysregulated host response to an infection (2).

Further deterioration can progress to septic shock, with a mortality rate of over 50%, with sepsis-related deaths being estimated to cause 1 in 5 of all deaths globally (3). Sepsis is costly; in U.S. hospitals, an average of $32,421 is spent per sepsis patient, resulting in an annual national cost of $20 billion (4). Timely and optimal antimicrobial therapy is key to improving sepsis patient outcomes and reducing costs. It is recommended that patients receive antibiotic treatment within 1 h of the diagnosis of sepsis (5). Still, the presence of multidrug-resistant (MDR) and extremely drug-resistant (XDR) Gram-negative bacteria is strongly associated with mortality (6, 7), partially due to inadequate initial therapy (8).

Empirical treatment is initiated with the use of broad-spectrum antibiotics. However, empirical therapy is ineffective in a portion of sepsis patients. Micek et al. found that empirical treatment was ineffective in 11.7% of early-onset sepsis and 23.6% of late-onset sepsis cases (9). A review by Marquet et al. demonstrated that there was a ≥50% incidence of ineffective empirical therapy for in-hospital severe infections reported in 13 of the 27 included papers (10). Antimicrobial susceptibility testing (AST) is imperative for determining optimal antimicrobial treatment, avoiding the overuse of broad-spectrum antimicrobials, and reducing side effects (11). The ISO standard for phenotypic AST is MIC determination using broth microdilution (BMD) (12). Most laboratories rely upon disc diffusion or automated methods calibrated to the ISO standard (13). Recently, rapid AST devices and methods have been developed to reduce the turnaround time (TAT) and allow optimal treatment to begin sooner than with the current methods used (11, 14).

ASTar (Q-linea AB, Uppsala, Sweden), is one of several new commercially available rapid phenotypic devices that automatically perform AST directly from positive blood culture bottles (BCBs). ASTar uses concentration determination to produce a controlled final inoculum according to EUCAST guidelines and has a broad Gram-negative panel of 336 testing wells in a compact disk (CD)-sized disc format. BMD and microscopy are used to generate MIC values. ASTar is not designed to identify microbial species. Species information needs to be provided to the system before results can be reported.

The aim of this study was to assess the performance of ASTar compared to BMD as part of susceptibility testing of Gram-negative isolates from positive blood cultures (PBCs). This study is the first performance assessment of the ASTar system and was designed based on ISO 20776-2:2007 requirements for *in vitro* devices (15). BMD using custom-made Sensititre plates (Thermo Fisher Scientific) was used as a reference method equivalent to ISO 20776-1:2019 (11).

Categorical agreement (CA), essential agreement (EA), and discrepancies were all assessed between the ASTar and Sensititre systems. Testing was performed across three sites, utilizing 412 contrived clinical isolates in human blood samples and 74 clinical patient samples. The ASTar BC G− (Gram-negative) kit panel contains 23 antimicrobials, including cefoxitin as a screening agent, testing *Enterobacterales*, Pseudomonas aeruginosa, Acinetobacter baumannii, and Haemophilus influenzae.

## MATERIALS AND METHODS

### ASTar system.

**FIG 1:**
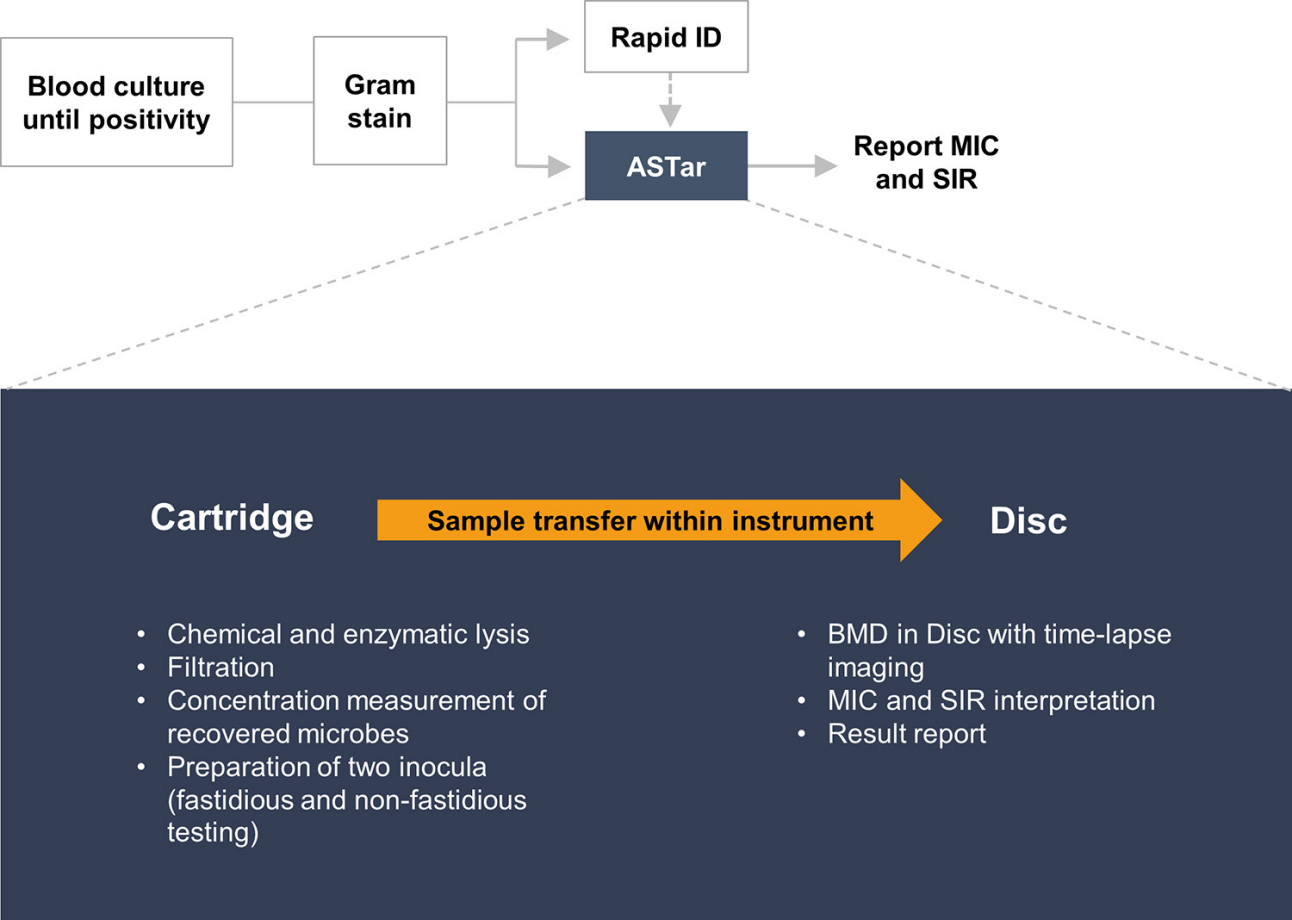
Example of the ASTar system’s clinical laboratory workflow. After blood culture positivity, Gram staining is performed. Next, a specimen can be loaded into the ASTar system for fully automated analysis (indicated in blue). In parallel, rapid identification (ID) can be performed with the laboratory’s standard methods. To start an ASTar run, a frozen insert should be placed into the cartridge along with a specimen from a Gram-negative, monomicrobial blood culture. The cartridge is scanned and loaded along with a disc into the instrument. The frozen insert contains all necessary reagents for the preparation of two inocula. Chemical and enzymatic lysis prepares the sample for filtration. The concentration of recovered microbes is then measured and adjusted to two inocula of 2 × 10^5^ to 8 × 10^5^ CFU/mL each. These are automatically pipetted onto the disc within the instrument, and BMD is performed on the disc, which contains antimicrobials at a range of 2-fold concentrations. Images from time-lapse microscopy are then analyzed with proprietary algorithms, and the MIC and category (SIR) are interpreted. The interpretation is dependent on species information, which should be provided at some point before results are reported.

The ASTar system consists of the ASTar instrument and the ASTar BC G− kit (Fig. 1). The instrument was run according to the manufacturer’s instructions, using the ASTar BC G− consumable kit, the ASTar BC G− frozen insert, and ASTar BC G− kit software. The ASTar BC G− consumable kit consists of the ASTar BC G− cartridge and disc. ASTar performs fully automated processing of a blood culture sample to report results on MICs and susceptible (S), intermediate (I), or resistant (R) (SIR) category. The technology utilized by ASTar is based on broth microdilution (BMD). In the ASTar system, bacteria are isolated from the blood culture via a combination of chemical and enzymatic lysis of nonmicrobial matter followed by filtration and the recovery of bacteria in cation-adjusted Mueller-Hinton broth (CAMHB). An aliquot of the recovered bacterial suspension is used for the determination of the concentration of bacteria. The recovered bacteria are diluted to a predetermined concentration suitable for the subsequent AST according to the ISO standard (12). One portion is diluted in CAMHB, and one portion is diluted in medium that supports the growth of fastidious bacteria, based on the concentration determination value (CAMHB supplemented with 0.02 g/L β-NAD and 5% lysed horse blood). Incubation takes place in the disc, which contains wells with antimicrobials at different concentrations. Bacterial growth is then monitored via time-lapse microscopy, and the result is interpreted via image analysis. When the species identity has been added to the system, the MIC and the associated SIR interpretation are reported. A run is performed by adding the blood culture sample to the ASTar BC G− cartridge and loading the cartridge into the ASTar instrument.

To allow the system to interpret the AST result, bacterial identity needs to be provided either at the start of, during, or after the run. Interpretation of the SIR category in this study was performed using the clinical breakpoints of EUCAST (version 11.0) (16). The study data were generated with ASTar BC G− kit software version 1.0, and performance analysis was performed with ASTar BC G− kit software version 1.5.

Quality control (QC) was performed with one of the four QC strains, sequentially, each day that blood culture samples were run. These strains were P. aeruginosa ATCC 27853, Escherichia coli ATCC 25922, Streptococcus pneumoniae ATCC 49619, and Klebsiella pneumoniae ATCC 700603. A culture of a QC strain grown overnight on an agar plate was then dissolved in medium and added to the sample inlet of a cartridge where ASTar measured the concentration and adjusted it to a predefined inoculum, followed by loading it into an ASTar disc.

### Clinical accuracy study.

**(i) Retrospective study arm.** The retrospective arm was performed at the Q-linea microbiology laboratory (Uppsala, Sweden). A study isolate bank of 415 study isolates had been collected and created from clinical laboratories in Örebro (Sweden), Hvidovre (Copenhagen, Denmark), Gävle (Sweden), and Bologna (Italy); the EUCAST development laboratory (Växjö, Sweden); and the Antibiotic Resistance Isolate Bank (Centers for Disease Control and Prevention [CDC], Atlanta, GA, USA), covering the following species typically identified by matrix-assisted laser desorption ionization–time of flight (MALDI-TOF) mass spectrometry: Escherichia coli, Klebsiella pneumoniae, Klebsiella oxytoca, Klebsiella aerogenes, Proteus mirabilis, Proteus vulgaris, Enterobacter cloacae, Citrobacter freundii, Citrobacter koseri, Serratia marcescens, Morganella morganii, Pseudomonas aeruginosa, Acinetobacter baumannii, and Haemophilus influenzae.

The isolates, stored at −80°C, were streaked onto UriSelect plates (Bio-Rad) for nonfastidious species and hematin (BD chocolate agar) agar for H. influenzae. A suspension at a 0.5 McFarland standard was prepared in a phosphate-buffered saline solution, and 5 μL was mixed with 9 mL of human blood containing sodium polyanethol sulfonate. The mixture was then inoculated into blood culture bottles, adding a blood volume according to the manufacturers’ instructions for the following bottles: Bactec Plus aerobic (Becton, Dickinson and Company), Bactec Peds Plus (Becton, Dickinson and Company), and BacT/Alert FA Plus aerobic and BacT/Alert PF Plus (bioMérieux). Incubation was performed to positivity in dedicated blood culture cabinets; for the contrived samples, either Bactec FX40 (Becton, Dickinson and Company) or BacT/Alert 3D (bioMérieux) was used.

Samples (1 mL) of positive blood culture suspensions were added to the cartridge of the ASTar BC G− kit. After scanning, the cartridge was then loaded along with the AST disc into the ASTar system, and the run was initiated. MIC and SIR results and images for each sample were saved on the instrument and then stored on a server. A purity check of the contrived blood cultures was performed by streaking blood culture samples onto UriSelect plates for all species except H. influenzae, for which hematin agar (BD chocolate agar) was used. The plates were inspected after incubation overnight.

**(ii) Prospective study arm.** The inclusion criteria for the prospective arm of the study were positive blood cultures of monobacterial, Gram-negative bacterial species characterized by rapid MALDI-TOF (Biotyper; Bruker) as the following: Acinetobacter baumannii, Citrobacter freundii, Citrobacter koseri, Enterobacter cloacae complex, Escherichia coli, Haemophilus influenzae, Klebsiella aerogenes, Klebsiella oxytoca, Klebsiella pneumoniae, Morganella morganii, Proteus mirabilis, Proteus vulgaris, Pseudomonas aeruginosa, and Serratia marcescens. Strains reported by MALDI-TOF as Raoultella ornithinolytica (*n* = 1) were included in the panel as K. oxytoca, and similarly, Klebsiella variicola (*n* = 3) strains were included in the panel as K. pneumoniae. Blood cultures were loaded into the ASTar system and run within 16 h of signaling positive in the blood culture cabinets, according to the instructions for use. The operators were instructed to load samples from the first eligible positive blood culture from a patient. Screening failures were defined as samples that had been loaded into the ASTar system but subsequently were found to not fulfill inclusion criteria.

At Uppsala University Hospital (Uppsala, Sweden) and Örebro University Hospital (Örebro, Sweden), between 3 March and 14 June 2021, positive BCBs were loaded into the ASTar system within 16 h of the bottle being flagged as positive in the blood culture incubator system, according to the instructions for use.

At Uppsala University Hospital, blood culturing was performed using the BacT/Alert Virtuo incubation cabinet in combination with BacT/Alert FA Plus aerobic, BacT/Alert PF Plus, and BacT/Alert FN Plus anaerobic blood culture bottles (bioMérieux). At Örebro University Hospital, blood cultures were performed using the Bactec blood culture cabinet with Bactec Plus aerobic and Bactec Peds Plus bottles (Becton, Dickinson and Company). The types of bottles used in the retrospective and prospective study arms are shown in Table 1. After Gram staining, 1-mL samples from the positive blood culture bottles were added to the ASTar BC G− kit cartridges. The kits were then loaded into the ASTar system. Data were stored in an external hard drive and analyzed with ASTar BC G− kit software version 1.5.

**TABLE 1:** Bottle types for the samples included in the study and their distribution over the two study arms

Bottle type	No. of samples		
	Retrospective arm	Prospective arm	Total
Bactec Peds Plus (plastic)	0	1	1
Bactec Peds Plus (glass)	32	0	32
Bactec Plus aerobic (plastic)	0	15	15
Bactec Plus aerobic (glass)	141	0	141
BacT/Alert FA Plus aerobic	216	31	247
BacT/Alert FN Plus anaerobic	0	25	25
BacT/Alert PF Plus	23	2	25

Species identification was performed by rapid MALDI-TOF analysis using the MALDI Biotyper system (Bruker) at both clinical laboratories. Identification was performed either from smear growth from incubation for 4 to 6 h on agar plates or from preparations by in-house protocols used at the respective hospitals. A score of 2.0 or higher from the MALDI Biotyper system was considered a valid result. A purity check of the blood cultures was performed by streaking blood culture samples onto UriSelect plates for all species except H. influenzae, for which hematin agar (BD chocolate agar) was used. The plates were inspected after incubation overnight. Frozen isolates from the clinical positive blood cultures were sent to Q-linea for characterization of the reference MICs.

The results obtained from the ASTar system were compared to the results of the reference method using BMD dry plates (Sensititre; Thermo Fisher Scientific), performed at the Q-linea microbiology laboratory (Uppsala, Sweden).

### Reference broth microdilution method.

Reference MICs were determined for all isolates, except for isolates from the CDC, at Q-linea using BMD. Isolates for BMD were cultured on agar plates, with nonfastidious isolates being cultured on tryptic soy agar (TSA) plates (Becton, Dickinson and Company) and UriSelect and H. influenzae being cultured on hematin test medium (BD chocolate agar), and incubated overnight. Custom-made Sensititre AST plates were used, and after incubation at 35°C for 16 to 20 h, plates were read using a mirror box. MIC values for the 66 antimicrobial-resistant (AR) isolates (corresponding to 13.6% of all isolates) from the CDC were downloaded on 17 September 2020 (17).

The isolates obtained from the CDC lacked reference MIC values for amoxicillin-clavulanic acid and cefuroxime. Therefore, results for 22 data points were added in conjunction with discrepancy resolution as these values were lacking. The added values were for amoxicillin-clavulanic acid (*n* = 10), cefuroxime (*n* = 10), ceftolozane-tazobactam (*n* = 1), and ceftazidime-avibactam (*n* = 1).

### Discrepancy analysis.

The ISO 20776-2:2007 definitions of the S, I, and R susceptibility testing categories do not match those of the most recent EUCAST clinical breakpoint updates (15). According to EUCAST clinical breakpoints of version 10 and onward, for several combinations, the S category has been lowered to 0.001 μg/mL, and thereby, in practice, the S category has been removed and replaced by I (susceptible with increased exposure) (16). This has implications for how very major discrepancies (VMDs) and major discrepancies (MDs) are calculated. VMDs and MDs can be calculated only when both the S and R categories are present. Therefore, according to the EUCAST version 11.0 clinical breakpoints, and still adhering to the ISO standard, neither MDs nor VMDs can be calculated for combinations lacking the “S” susceptibility testing category. Therefore, two alternative strategies were used for discrepancy calculations. In strategy 1, MDs and VMDs were interpreted according to the ISO standard followed during the study and are presented in Table 2 (15). In strategy 1, no MDs and VMDs were calculated for combinations that lacked the S category. For some antimicrobials such as cefuroxime and cefazolin, this concerns all tested species. In other cases, such as for ciprofloxacin, this applies to P. aeruginosa and A. baumannii but not *Enterobacterales*. In strategy 2, for combinations lacking an S category designation, VMDs and MDs were calculated as if all intermediate results were interpreted as susceptible, and therefore, higher percent discrepancies are reported for certain combinations (see Table 2). According to the ISO standard, discrepancies can be investigated by performing triplicate BMD.

**TABLE 2:** Accuracy study results for each antimicrobial, including contrived and clinical samples, after discrepancy resolution^*g*^

Antimicrobial agent	EA (%)	CA (%)	VMDs (%)	MDs (%)
Ampicillin	233/241 (96.7)	237/241 (98.3)	1/99 (1.0)	3/142 (2.1)
Amoxicillin-clavulanic acid^*a*^	341/357 (95.5)	332/357 (93.0)	4/79 (5.1)	21/278 (7.6)
Piperacillin-tazobactam^*b*^	416/436 (95.4)	426/436 (97.7)	5/69 (7.2)^*f*^	4/354 (1.1)^*f*^
Cefazolin	276/286 (96.5)	262/286 (91.6)	0/121 (0)^*f*^	NA^*f*^
Cefepime	440/452 (97.3)^*e*^	435/441 (98.6)^*e*^	0/55 (0)^*f*^	0/367 (0)^*f*^
Cefotaxime	422/443 (95.3)	438/443 (98.9)	0/61 (0)	4/380 (1.1)
Ceftazidime	389/399 (97.5)	387/399 (97.0)	0/68 (0)^*f*^	3/310 (1.0)^*f*^
Ceftazidime-avibactam^*c*^	393/429 (91.6)	422/429 (98.4)	1/10 (10.0)	6/419 (1.4)
Ceftolozane-tazobactam^*b*^	416/426 (97.7)	418/426 (98.1)	3/38 (7.9)	5/388 (1.3)
Ceftriaxone	429/444 (96.6)	440/444 (99.1)	0/62 (0)	1/382 (0.3)
Cefuroxime	282/294 (95.9)	285/294 (96.9)	0/44 (0)^*f*^	NA^*f*^
Ertapenem	391/413 (94.7)	412/413 (99.8)	1/39 (2.6)	0/374 (0)
Meropenem	455/481 (94.6)	461/481 (95.8)	0/28 (0)	0/432 (0)
Aztreonam	421/427 (98.6)	421/427 (98.6)	1/68 (1.5)^*f*^	0/345 (0)^*f*^
Ciprofloxacin	431/447 (96.4)	429/447 (96.0)	0/88 (0)^*f*^	0/333 (0)^*f*^
Levofloxacin	466/475 (98.1)	459/475 (96.6)	1/80 (1.2)^*f*^	1/375 (0.3)^*f*^
Amikacin	413/448 (92.2)	442/448 (98.7)	3/20 (15.0)	3/428 (0.7)
Gentamicin	412/431 (95.6)	423/431 (98.1)	8/48 (16.7)	0/383 (0)
Tobramycin	428/451 (94.9)	448/451 (99.3)	1/61 (1.6)	2/390 (0.5)
Tigecycline	189/196 (96.4)	195/196 (99.5)	0/1 (0)	1/195 (0.5)
Colistin	237/251 (94.4)	251/251 (100)	0/13 (0)	0/238 (0)
Trimethoprim-sulfamethoxazole^*d*^	403/423 (95.3)	410/423 (96.9)	1/87 (1.1)	8/332 (2.4)

Total	8,283/8,650 (95.8)	8,433/8,639 (97.6)	30/1,239 (2.4)	62/6,845 (0.9)

a The concentration of clavulanic acid is fixed at 2 μg/mL.

b The concentration of tazobactam is fixed at 4 μg/mL.

c The concentration of avibactam is fixed at 4 μg/mL.

d Trimethoprim-sulfamethoxazole at a ratio of 1:19.

e There is no clinical breakpoint for A. baumannii and cefepime; thus, EA but not CA could be calculated.

f VMD and MD calculations were based on ISO 20776-2:2007, including antimicrobials without an S category (see EUCAST comments on arbitrary S breakpoints [16]). Alternative evaluation criteria could be applied where, in practice, there was no S category for one or more species (only I or R) (strategy 2). If all discrepancies in such cases are instead classified as either MDs or VMDs, the alternative results are as follows: 5/367 (1.4%) MDs and 5/69 (7.2%) VMDs for piperacillin-tazobactam, 19/165 (11.5%) MDs and 5/121 (4.1%) VMDs for cefazolin, 0/381 (0%) MDs and 0/55 (0%) VMDs for cefepime, 3/323 MDs (0.9%) and 0/68 (0%) VMDs for ceftazidime, 5/250 (2.0%) MDs and 4/44 (9.1%) VMDs for cefuroxime, 0/357 (0%) MDs and 1/68 (1.5%) VMDs for aztreonam, 0/350 (0%) MDs and 2/88 (2.3%) VMDs for ciprofloxacin, and 1/383 (0.3%) MDs and 2/80 (2.5%) VMDs for levofloxacin.

g Analysis was performed using ASTar BC G− kit software version 1.5, and the results were interpreted according to discrepancy calculation strategy 1. NA (not applicable) indicates that the percentage could not be calculated since, in practice, there is no susceptible category.

Such discrepancy resolution testing was performed for a subset of MDs and VMDs by performing BMD in triplicates for the deviating combinations. This approach could lead to the demonstration of lower possible EA and CA values than if all discrepant results would have been rerun. The discrepancy analysis was performed at the Q-linea laboratory using the same method and equipment as those used for the reference MIC characterizations. According to ISO 20776-2:2007, at least two of the three results should give the same category agreement (15). Moreover, all three repeat reference MIC values should be within a three-doubling-dilution interval of each other. Based on this, eight data points were removed from the data set. Data from discrepancy resolution testing are summarized in Table S1 in the supplemental material.

### Reproducibility assessment.

This study assessed the reproducibility of the ASTar system results using contrived samples, prepared according to the methods described above for the retrospective study arm. Positive blood cultures were prepared from 11 different isolates of Escherichia coli, Enterobacter cloacae, Haemophilus influenzae, Klebsiella aerogenes, Klebsiella pneumoniae, and Pseudomonas aeruginosa with BacT/Alert FA Plus aerobic bottles (bioMérieux). Triplicate samples from each bottle were run on three different ASTar instruments (Q-linea, Uppsala, Sweden). The procedure was repeated over 2 days. The best-case calculation for reproducibility assumed that any off-scale results were within 1 dilution from the adjacent on-scale result. The worst-case calculation assumed that any off-scale results were more than 1 dilution from the adjacent on-scale result.

### Ethics.

For patient samples, only leftover specimens that would otherwise have been discarded were used. No patient data were collected that could link a patient sample to a participant’s identity. This ensured that anonymity was maintained. The hospital laboratories approved this procedure. Contrived blood cultures were prepared with human blood, which was collected under an application approved by the ethical review board, Uppsala, Sweden (application reference number 2020-00560). Informed consent was obtained from participants providing blood for the contrived samples.

## RESULTS

**FIG 2:**
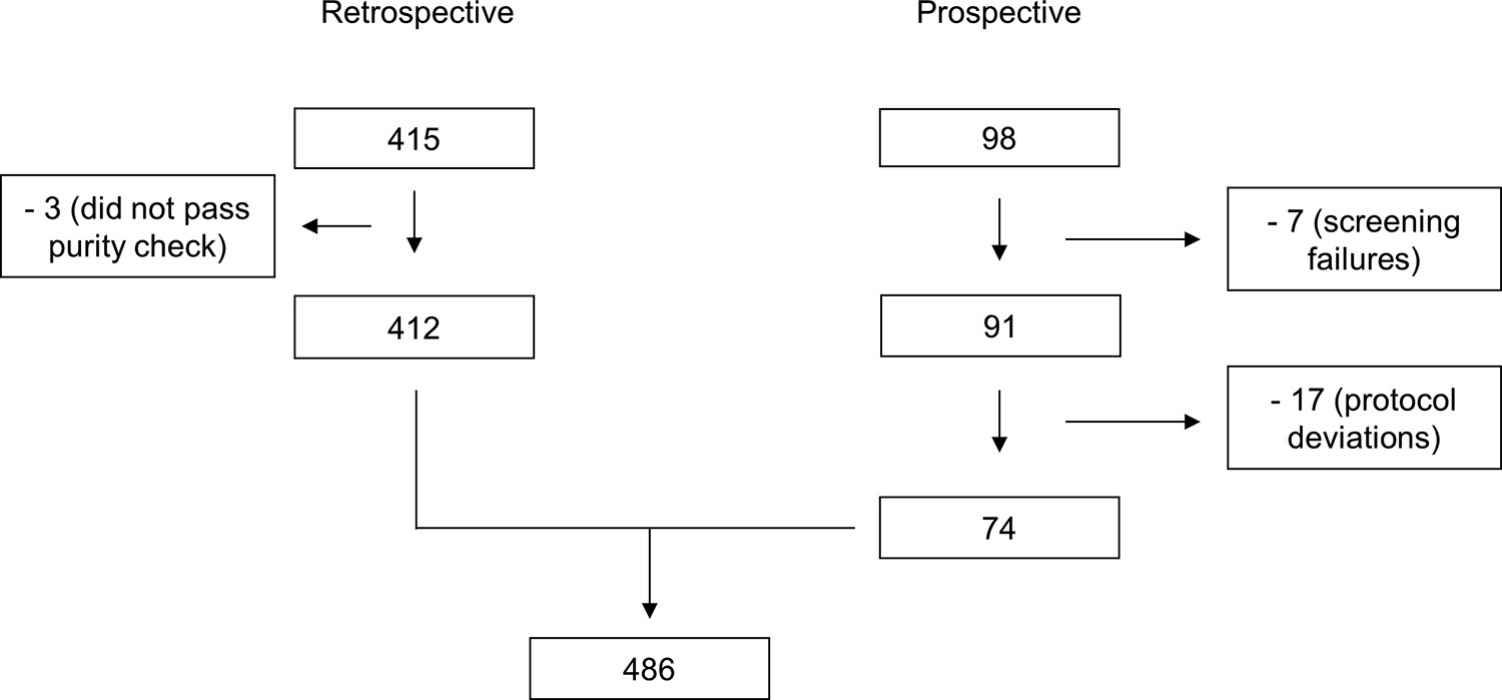
Overview of the included and excluded isolates within the two study arms.

A total of 486 isolates could be included in the study, with the retrospective arm of the study accounting for 412 isolates after the exclusion of 3 isolates verified to contain polymicrobial samples. The prospective arm of the study screened a total of 98 samples. Of these, 74 could be included, with 58 samples from Uppsala University Hospital and 16 from Örebro University Hospital. In total, seven screening failures were recorded, and these were withdrawn from the study data set. Three of the screening failures were polymicrobial samples, three were off-panel species (Parabacteroides distasonis, Pseudomonas koreensis, and Stenotrophomonas maltophilia), and one lacked species information. Deviations from the protocol led to the withdrawal of 17 clinical samples (Fig. 2). The evaluated bacterial species and the number of strains tested within the two arms of the study are summarized in Table 3.

**TABLE 3:** Bacterial species and numbers of isolates tested within the two arms of the study

Bacterial species	No. of isolates		
	Prospective arm	Retrospective arm	Total
Acinetobacter baumannii	1	10	11
Citrobacter freundii	1	10	11
Citrobacter koseri	2	19	21
Enterobacter cloacae complex	4	15	19
Escherichia coli	44	131	175
Haemophilus influenzae	0	28	28
Klebsiella aerogenes	1	10	11
Klebsiella oxytoca ^*a*^	5	25	30
Klebsiella pneumoniae ^*b*^	13	70	83
Morganella morganii	0	10	10
Proteus mirabilis	1	39	40
Proteus vulgaris	0	14	14
Pseudomonas aeruginosa	2	21	23
Serratia marcescens	0	10	10

Total	74	412	486

a The sample characterized by MALDI-TOF analysis as Raoultella ornithinolytica was included as K. oxytoca (*n* = 1).

b The samples characterized by MALDI-TOF analysis as Klebsiella variicola (*n* = 3) were included as K. pneumoniae.

ASTar generated 8,650 accuracy data points from a total of 14 fastidious and nonfastidious Gram-negative bacterial species. Performance data are shown in Table 2. Comparison between ASTar and the reference method revealed an overall EA of 95.8% (8,283/8,650) and a CA of 97.6% (8,433/8,639). The denominator difference between EA and CA is explained by one combination for which the MIC was determined and presented but where there was no clinical breakpoint (A. baumannii for cefepime) (14). The reference MIC distributions for the strains included in this study are shown in Fig. S1 in the supplemental material.

According to strategy 1, the overall MD rate was 0.9% (62/6,845), and the VMD rate was 2.4% (30/1,239). Of these, 31/62 MDs and 17/30 VMDs were in essential agreement with the reference MICs. For discrepancies (31 MDs and 13 VMDs) not in essential agreement with the reference MICs, the VMDs for amikacin (1/20), ceftazidime-avibactam (1/10), and amoxicillin-clavulanic acid (3/79) were measured as >3%, whereas no MDs of >3% were measured.

**FIG 3:**
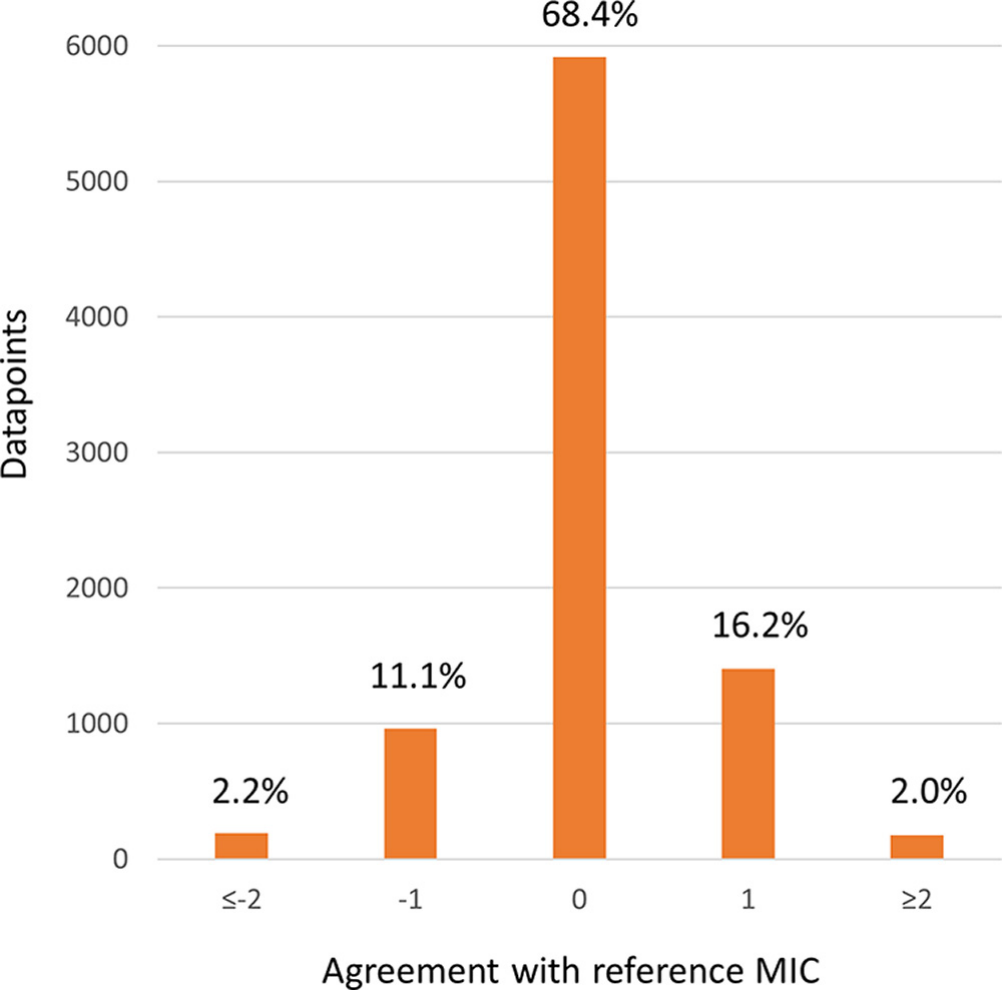
Accuracy of the ASTar MIC compared to the reference MIC, with the number of doubling dilutions from the reference MIC. In most cases, the reference MIC range covers or exceeds the full ASTar range. For a subset (6.6%) where reference MICs do not cover the full range of the MICs of the ASTar BC G^−^ kit, the comparisons of ASTar MIC ranges have been shortened to be comparable with the reference MIC ranges.

The agreement distribution between the ASTar and reference MICs is outlined in Fig. 3. Figure S2 shows the individual MDs for *Enterobacterales*, P. aeruginosa, A. baumannii, and H. influenzae. Figure S3 shows the individual VMDs for *Enterobacterales*, P. aeruginosa, and A. baumannii. No VMDs were recorded for H. influenzae.

Cefoxitin was used to screen for AmpC-producing *Enterobacterales*, determined as values higher than the epidemiological cutoff (ECOFF) value of 8 μg/mL. Performance was evaluated using BMD reference MICs (14). The positive agreement and negative agreement exceeded 90%, as shown in Table S2. Cefoxitin results that were in nonagreement with the reference results are shown in Table S3.

Table 4 shows the percentages of the study isolates with reference MICs that were within ±1 dilution from the clinical breakpoints.

**TABLE 4:** Proportions of isolates with reference MICs close to the clinical breakpoint for the tested antimicrobials^*a*^

Antimicrobial agent	No. of isolates with MIC within ±1 doubling dilution of the CBP	Total no. of isolates	% of isolates with MIC within ±1 doubling dilution of the CBP
Amikacin	111	448	25
Amoxicillin-clavulanic acid	173	357	48
Ampicillin	80	241	33
Aztreonam	38	427	9
Cefazolin	117	286	41
Cefepime	29	441	7
Cefotaxime	35	443	8
Cefoxitin	239	325	74
Ceftazidime	103	399	26
Ceftazidime-avibactam	15	429	3
Ceftolozane-tazobactam	43	426	10
Ceftriaxone	29	444	7
Cefuroxime	106	294	36
Ciprofloxacin	56	447	13
Colistin	35	251	14
Ertapenem	12	413	3
Gentamicin	130	431	30
Levofloxacin	61	475	13
Meropenem	32	481	7
Piperacillin-tazobactam	113	436	26
Tigecycline	109	196	56
Tobramycin	180	451	40
Trimethoprim-sulfamethoxazole	17	423	4

a See reference 16. CBP, clinical breakpoint.

In addition to accuracy, the study also evaluated the reproducibility of ASTar results (Table S4), which was shown to be 99.5% for a best-case calculation and 97.5% for a worst-case calculation. Reproducibility was >98% for 22 of 23 antimicrobials. For meropenem, reproducibility was measured as 89.7% for both best-case and worst-case calculations, driven by a single E. cloacae isolate that had a significant impact on the results.

## DISCUSSION

ASTar provides MIC values for testing a broad panel across wide concentration ranges, with 23 antimicrobials for nonfastidious Gram-negative bacteria and 6 antimicrobials for fastidious Gram-negative bacteria. The performance of the ASTar BC G− kit using version 1.5 software was evaluated against BMD (Sensititre). Acceptance criteria in this study were defined for *in vitro* devices according to ISO 20776-2:2007. The ASTar system delivered results with an EA of 95.8% and a CA of 97.6%. The overall MD rate was 0.9%, and the overall VMD rate was 2.4%. Of the measured categorical discrepancies, 52% were in essential agreement. Reproducibility was shown to be high, >98% for 22 of 23 antimicrobials.

This study had several strengths. First, the study included two geographically distinct clinical sites (Örebro and Uppsala), allowing the performance of the ASTar system to be tested with clinical isolates from patients with confirmed BSI. Contrived samples were used to supplement clinical samples, including isolates that were resistant or close to clinical breakpoints in the data set. Second, the study data set had a wide range of MICs across the included isolates, as shown in Fig. S1 in the supplemental material. This is possible due to the breadth of 2-fold concentration dilutions present in the ASTar disc for the antimicrobials. Finally, a high number of isolates had MICs close to clinical breakpoints. Table 4 shows the percentage of MICs that were within ±1 dilution from the clinical breakpoints. For eight of the antimicrobials in the study, ≥30% of all MICs were within ±1 dilution from the clinical breakpoints. This proximity to the clinical breakpoints compared to the reference MIC may result in categorical discrepancies, and essential agreement under these circumstances may be a better measure of performance. Of the measured discrepancies, 52% were in essential agreement.

In instances where the testing does not include a significant number of resistant isolates, any error has a large impact on VMD calculations. In these cases, EA could be a better measure of performance. The tested number of resistant isolates in the study was related to the prevalence of resistant isolates, which was limited for certain antimicrobials. For instances where there are fewer than 34 resistant isolates, individual errors will then be reflected as a VMD rate of ≥3% (15). This effect could be seen for both ceftazidime-avibactam and amikacin. Similarly, for combinations lacking an intermediate category, EA could give a better estimate of the performance of that antimicrobial. Antimicrobials for which there is no intermediate category include amikacin, piperacillin-tazobactam, ceftolozane-tazobactam, gentamicin, and amoxicillin-clavulanic acid.

There were also some limitations of the present study. First, both clinical sites were located in Sweden, which has not had the same emergence of resistant bacteria as other European countries in recent decades (18). This limitation was offset with contrived resistant samples to generate a sample pool with a good mixture of resistant and susceptible isolates as well as a variety of MIC values (Fig. S1). Second, although blood culture bottles from both bioMérieux and Becton, Dickinson and Company were used in the study (Table 1), the anaerobic plastic bottle (Bactec Plus anaerobic/F, catalog number 442022) used at the Örebro (Sweden) site in clinical routine was not included in the ASTar BC G− kit’s instructions for use. Therefore, samples of positive anaerobic blood culture bottles from the Örebro site were not included in this study.

The increasing prevalence of MDR and XDR bacteria poses a threat to the continued effectiveness of antimicrobial treatment. Rapid AST allows faster optimal treatment and faster de-escalation of empirical, broad-spectrum treatment than with conventional AST methods. The continued and ineffective use of broad-spectrum antimicrobials leads to higher patient care costs and exposes patients to unnecessary toxicity (19).

This study was not designed to assess the clinical outcomes, laboratory workflow, or health economic benefits of the ASTar system. The assessment of the performance of the ASTar system in these settings is an area for future research. Ineffective empirical therapy exposes patients to unnecessary toxicity and is associated with higher mortality rates (8). Reducing the time to obtain AST results reduces the time for patients to receive optimal therapy. While it is expected that rapid AST devices can decrease patient mortality, further studies are needed to demonstrate this (20). However, rapid AST has been shown in other studies to decrease the length of stay and hospitalization costs (20, 21).

This study demonstrated that the ASTar system performs reliably across a range of Gram-negative bacterial species and antimicrobials. These included the Gram-negative species that most commonly cause sepsis, such as Escherichia coli, Klebsiella spp., and Pseudomonas aeruginosa (22). The selection of study isolates aimed to include those where the reference MIC is close to a breakpoint (Table 4 and Fig. S1). In addition, 66 isolates from the CDC library were also included. The ASTar system delivered reproducible results with overall EA and CA of >95%, which, together with automation, is promising to support a simplified laboratory workflow and rapid support for patient antimicrobial treatment choices.
