# Supplementary material for: Performance of a System for Rapid Phenotypic Antimicrobial Susceptibility Testing of Gram-Negative Bacteria Directly from Positive Blood Culture Bottles
Source: J Clin Microbiol. 2023 Feb 28;61(3):e01525-22. doi: 10.1128/jcm.01525-22 (PMC10035315; doi:10.1128/jcm.01525-22)
Supplement: Supplemental file 1 — Supplemental material. Download jcm.01525-22-s0001.pdf, PDF file, 1.1 MB [file jcm.01525-22-s0001.pdf]

Supplementary data

Supplementary Table 1. Table of discrepancy resolution, with reference MIC before and after repeated BMD.

			Reference data at study start			Reference data after discrepancy resolution		
**Species**	**Isolate**	**Antimicrobial**	**Reference MIC**		**Category**	**Reference MIC**		**Category**
A. baumannii	HVI078	Amikacin	=	4	S	=	4	S
A. baumannii	HVI078	Gentamicin	=	4	S	=	8	R
A. baumannii	QM556	Ciprofloxacin	>	4	R	>	4	R
A. baumannii	QM556	Levofloxacin	>	4	R	>	4	R
A. baumannii	QM560	Gentamicin	=	1	S	=	1	S
A. baumannii	QM560	Meropenem	=	16	R	=	0,25	S
A. baumannii	QM561	Ciprofloxacin	>	16	R	=	0,5	I
A. baumannii	QM561	Levofloxacin	>	4	R	N/A		
C. freundii	HVI085	Ceftolozane-tazobactam	=	8	R	N/A		
C. freundii	HVI085	Piperacillin-tazobactam	=	32	R	=	32	R
C. koseri	ORE023	Amikacin	>	256	R	=	0,5	S
E. cloacae	AR0132	Meropenem	=	16	R	=	8	I
E. cloacae	AR0136	Meropenem	>	32	R	=	8	I
E. cloacae	HVI005	Ceftolozane-tazobactam	=	0,25	S	=	0,125	S
E. coli	AR0370	Amikacin	=	8	S	=	8	S
E. coli	AR0434	Trimethoprim-sulfamethoxazole	>	8	R	=	16	R
E. coli	AR0436	Ceftolozane-tazobactam	>	64	R	=	4	R
E. coli	AR0436	Gentamicin	=	4	R	=	2	S
E. coli	AR0436	Tigecycline	=	1	R	=	0,25	S
E. coli	AR0437	Ceftazidime-avibactam	=	8	S	=	8	S
E. coli	AR0437	Trimethoprim-sulfamethoxazole	>	32	R	=	2	S
E. coli	AR0450	Ceftazidime-avibactam	>	16	R	>	32	R
E. coli	HVI181	Amoxicillin-clavulanic acid	=	8	S	=	8	S
E. coli	HVI181	Cefuroxime	=	8	I	=	16	R
E. coli	HVI189	Piperacillin-tazobactam	=	4	S	=	4	S
E. coli	HVI190	Amoxicillin-clavulanic acid	=	8	S	=	8	S
E. coli	HVI196	Amoxicillin-clavulanic acid	=	8	S	N/A		
E. coli	HVI198	Tobramycin	=	4	R	=	0,5	S
E. coli	HVI212	Amoxicillin-clavulanic acid	=	8	S	=	8	S
E. coli	HVI232	Amoxicillin-clavulanic acid	=	8	S	=	8	S
E. coli	HVI236	Cefazolin	=	8	R	=	4	I
E. coli	HVI236	Ampicillin	=	16	R	=	16	R
E. coli	ORE069	Amoxicillin-clavulanic acid	=	8	S	=	8	S
E. coli	ORE070	Cefazolin	=	16	R	=	8	R

E. coli	ORE070	Levofloxacin	=	0,5	S	=	0,5	S
E. coli	ORE081	Amoxicillin-clavulanic acid	=	8	S	=	8	S
E. coli	ORE098	Cefuroxime	=	8	I	=	4	I
E. coli	ORE104	Amoxicillin-clavulanic acid	=	8	S	=	8	S
E. coli	ORE109	Amoxicillin-clavulanic acid	=	8	S	=	4	S
E. coli	ORE110	Trimethoprim-sulfamethoxazole	>	64	R	>	64	R
E. coli	ORE123	Amoxicillin-clavulanic acid	=	8	S	=	8	S
E. coli	ORE129	Amoxicillin-clavulanic acid	=	8	S	=	8	S
E. coli	ORE129	Cefuroxime	=	8	I	N/A		
E. coli	ORE135	Amoxicillin-clavulanic acid	=	8	S	=	8	S
E. coli	QM565	Amoxicillin-clavulanic acid	=	8	S	=	8	S
E. coli	QM565	Amikacin	=	1	S	=	1	S
H. influenzae	HVI010	Meropenem	<=	0,03	S	=	0,03	S
K. aerogenes	HVI116	Amikacin	=	64	R	=	2	S
K. aerogenes	ORE029	Aztreonam	>	256	R	=	0,06	S
K. oxytoca	AR0028	Cefazolin	>	32	R	=	8	R
K. oxytoca	HVI261	Cefazolin	=	16	R	=	8	R
K. pneumoniae	AR0010	Amikacin	=	8	S	=	8	S
K. pneumoniae	AR0010	Gentamicin	=	2	S	N/A		
K. pneumoniae	AR0066	Amikacin	=	32	R	=	8	S
K. pneumoniae	AR0076	Ertapenem	=	1	R	=	2	R
K. pneumoniae	AR0364	Trimethoprim-sulfamethoxazole	>	8	R	>	64	R
K. pneumoniae	AR0376	Ertapenem	=	0,25	S	N/A		
K. pneumoniae	HVI281	Cefazolin	=	8	R	=	2	I
K. pneumoniae	ORE142	Cefuroxime	=	16	R	=	2	I
K. pneumoniae	ORE144	Cefuroxime	=	16	R	=	8	I
K. pneumoniae	ORE146	Piperacillin-tazobactam	=	32	R	=	16	R
K. pneumoniae	ORE154	Piperacillin-tazobactam	>	256	R	N/A		
K. pneumoniae	ORE154	Cefazolin	>	64	R	=	16	R
K. pneumoniae	ORE171	Ceftolozane-tazobactam	=	8	R	=	2	S
K. pneumoniae	ORE171	Piperacillin-tazobactam	=	32	R	=	8	S
K. pneumoniae	QM843	Trimethoprim-sulfamethoxazole	=	0,5	S	=	0,5	S
K. pneumoniae	QM845	Trimethoprim-sulfamethoxazole	=	0,5	S	=	1	S
M. morganii	AR0519	Amikacin	=	16	R	=	8	S
M. morganii	AR0519	Ceftazidime-avibactam	<=	0,5	S	=	0,5	S
M. morganii	AR0519	Cefepime	=	1	S	=	2	I
M. morganii	HVI331	Cefotaxime	>	8	R	N/A		
M. morganii	HVI331	Tobramycin	=	8	R	=	8	R
M. morganii	HVI332	Cefotaxime	=	0,5	S	=	0,25	S
P. aeruginosa	AR0233	Cefepime	=	8	I	=	4	I
P. aeruginosa	AR0233	Piperacillin-tazobactam	=	16	I	=	16	I

P. aeruginosa	AR0233	Ciprofloxacin	=	1	R	=	0,5	I
P. aeruginosa	AR0233	Levofloxacin	=	4	R	=	2	R
P. aeruginosa	AR0257	Cefepime	=	8	I	=	8	I
P. aeruginosa	AR0262	Ciprofloxacin	=	2	R	=	8	R
P. aeruginosa	AR0445	Amikacin	=	64	R	=	64	R
P. aeruginosa	AR0512	Ciprofloxacin	=	2	R	=	1	R
P. aeruginosa	AR0512	Ceftazidime-avibactam	=	8	S	=	4	S
P. aeruginosa	AR0512	Cefepime	=	8	I	=	4	I
P. aeruginosa	AR0512	Ceftazidime	=	8	I	=	8	I
P. mirabilis	AR0377	Ertapenem	=	4	R	=	0,03	S
P. mirabilis	BOL043	Trimethoprim-sulfamethoxazole	=	1	S	=	1	S
P. mirabilis	HVI360	Gentamicin	=	4	R	=	2	S
P. mirabilis	HVI367	Trimethoprim-sulfamethoxazole	=	1	S	=	2	S
P. mirabilis	HVI370	Amikacin	=	8	S	=	8	S
P. mirabilis	HVI373	Gentamicin	=	4	R	=	1	S
P. mirabilis	HVI374	Gentamicin	=	0,5	S	=	0,5	S
P. mirabilis	HVI380	Trimethoprim-sulfamethoxazole	=	2	S	=	0,5	S
P. mirabilis	ORE262	Aztreonam	<=	0,03	S	<=	0,03	S
P. mirabilis	ORE262	Ceftolozane-tazobactam	=	0,5	S	=	0,5	S
P. vulgaris	HVI387	Ceftazidime	<=	0,25	S	=	0,06	S
P. vulgaris	HVI387	Cefotaxime	=	0,03	S	<=	0,016	S
P. vulgaris	HVI387	Trimethoprim-sulfamethoxazole	<=	0,06	S	=	0,06	S
S. marcescens	ORE058	Tobramycin	=	4	R	=	2	S

* For results denoted as N/A, no mode value could be obtained. Since a comparison between ASTar and the reference method cannot be performed for these datapoints, these datapoints have been removed in accordance with the ISO standard 20776-2:2007 (1).

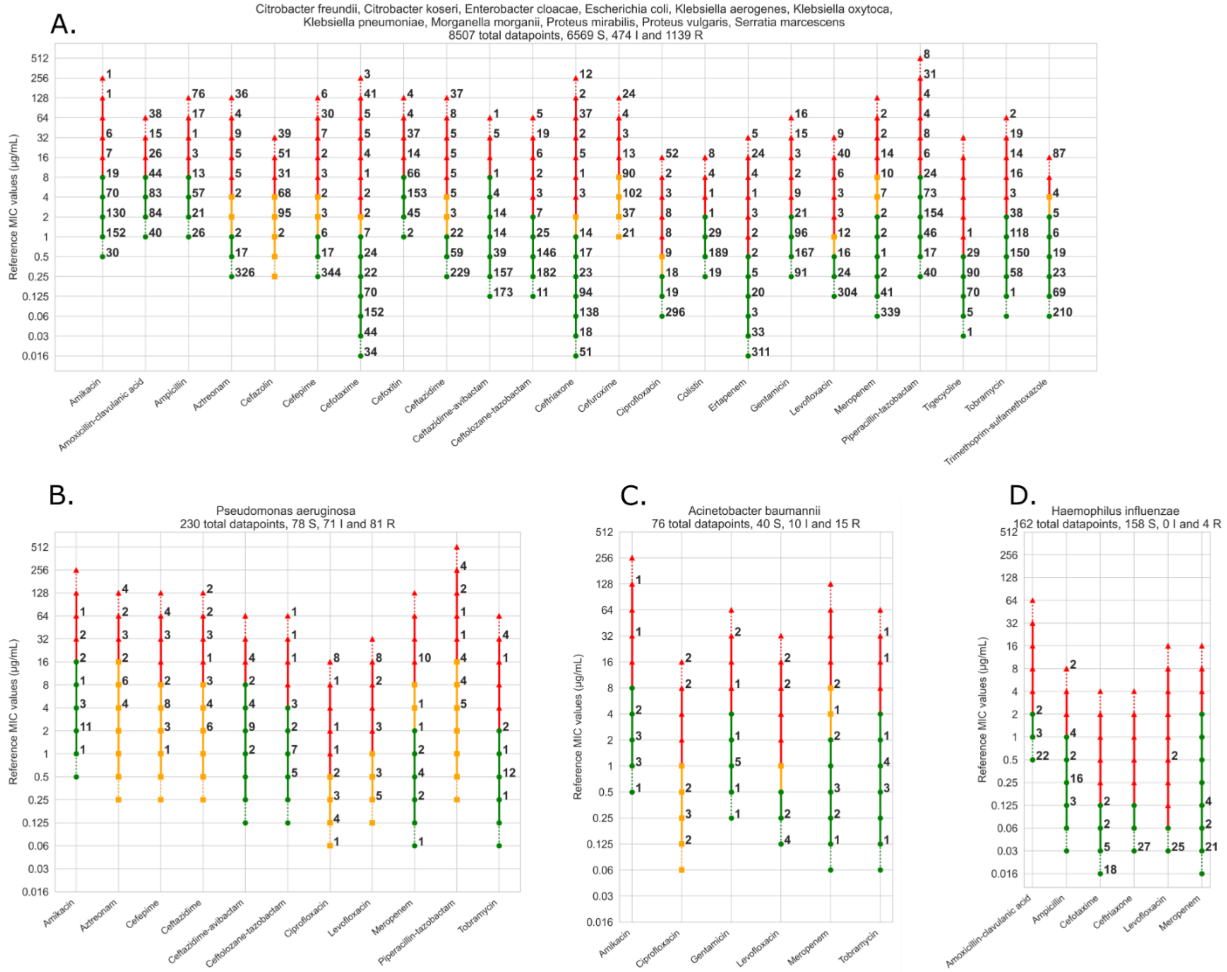

Supplementary Figure 1. The distribution of the reference MICs for the included study strains displayed along the reportable antimicrobial ranges of the ASTar BC G- Kit. Where reference MIC ranges were broader than those of ASTar, they were truncated to ASTar ranges. In some instances, reference MIC range was shorter than that of ASTar, primarily concerning CDC isolates. The numbers indicate the number of strains with a given reference MIC. Off-scale reference MICs are indicated as dashed lines (≤ and >), e.g. ASTar BC G- Kit reportable range for amikacin is 0.5 – 128 µg/mL. Green indicates ‘S’, yellow ‘I’, and red ‘R’.

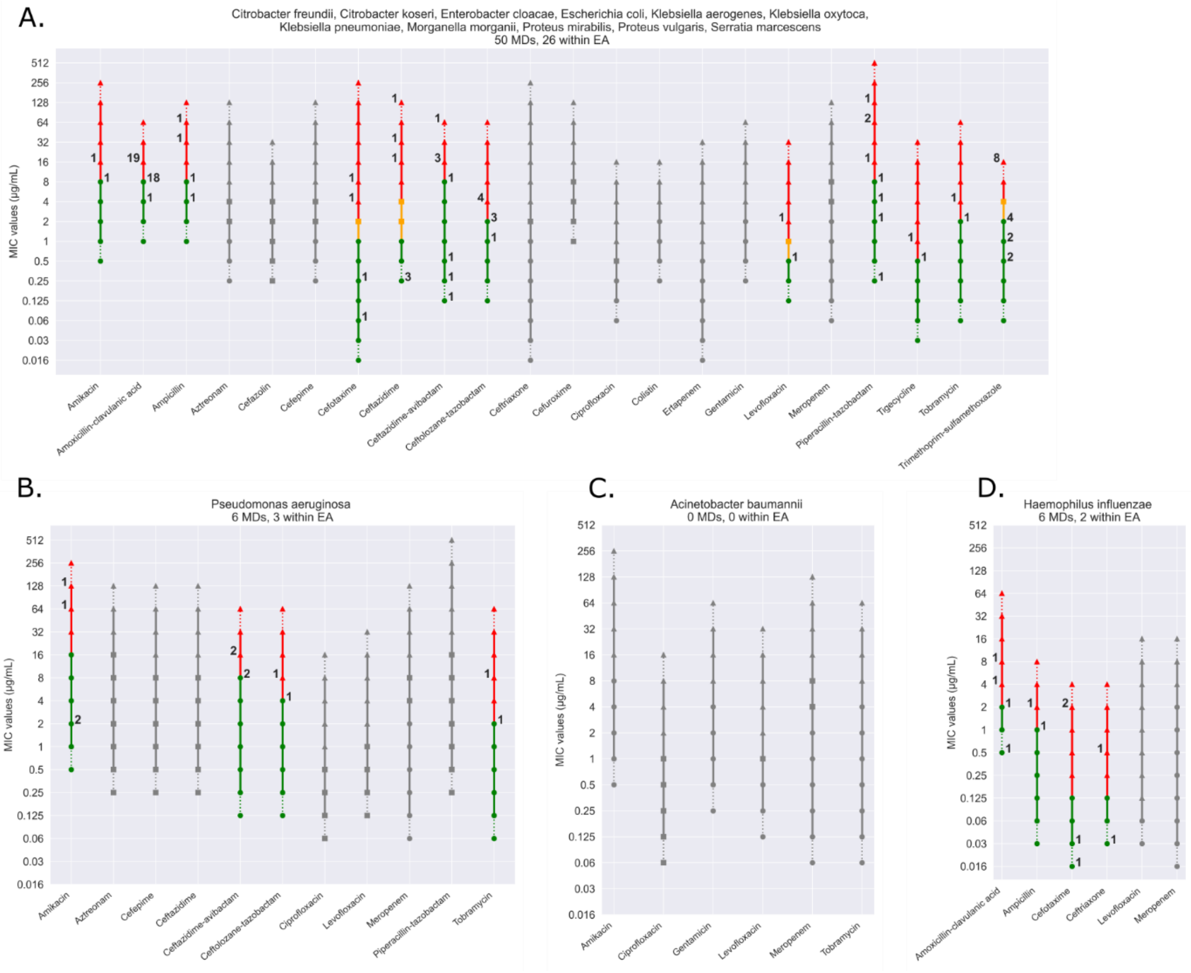

Supplementary Figure 2.

Major discrepancy results from analysis using ASTar BC G- Kit software version 1.5 and interpreted according to discrepancy calculation strategy 1 and after discrepancy resolution. Where reference MIC ranges were broader than those of ASTar, they were truncated to ASTar ranges. In some instances reference MIC range was shorter than that of ASTar, primarily concerning CDC isolates. ASTar MIC results are presented to the left of the range line and corresponding reference MIC values are indicated to the right. Green indicates ‘S’, yellow ‘I’, and red ‘R’.

A. Enterobacterales

Major discrepancies for Enterobacterales of more than one MIC value from reference MIC (non EA) were 1 amoxicillin-clavuanic acid, 2 ampicillin, 2 cefotaxime, 3 ceftazidime, 3 ceftazidime-avibactam, 1 ceftolozane-tazobactam, 1 levofloxacin, and 3

piperacillin-tazobactam.

B. *P. aeruginosa*

Major discrepancies for *P. aeruginosa* of more than one MIC value from reference MIC (non EA) were 2 amikacin and 1 tobramycin.

C. *A. baumannii*

No major discrepancies were registered for *A. baumannii*.

D. *H. influenzae*

Major discrepancies for *H. influenzae* of more than one MIC value from reference MIC (non EA) were 1 ceftriaxone, 2 cefotaxime, and 1 amoxicillin-clavulanic acid.

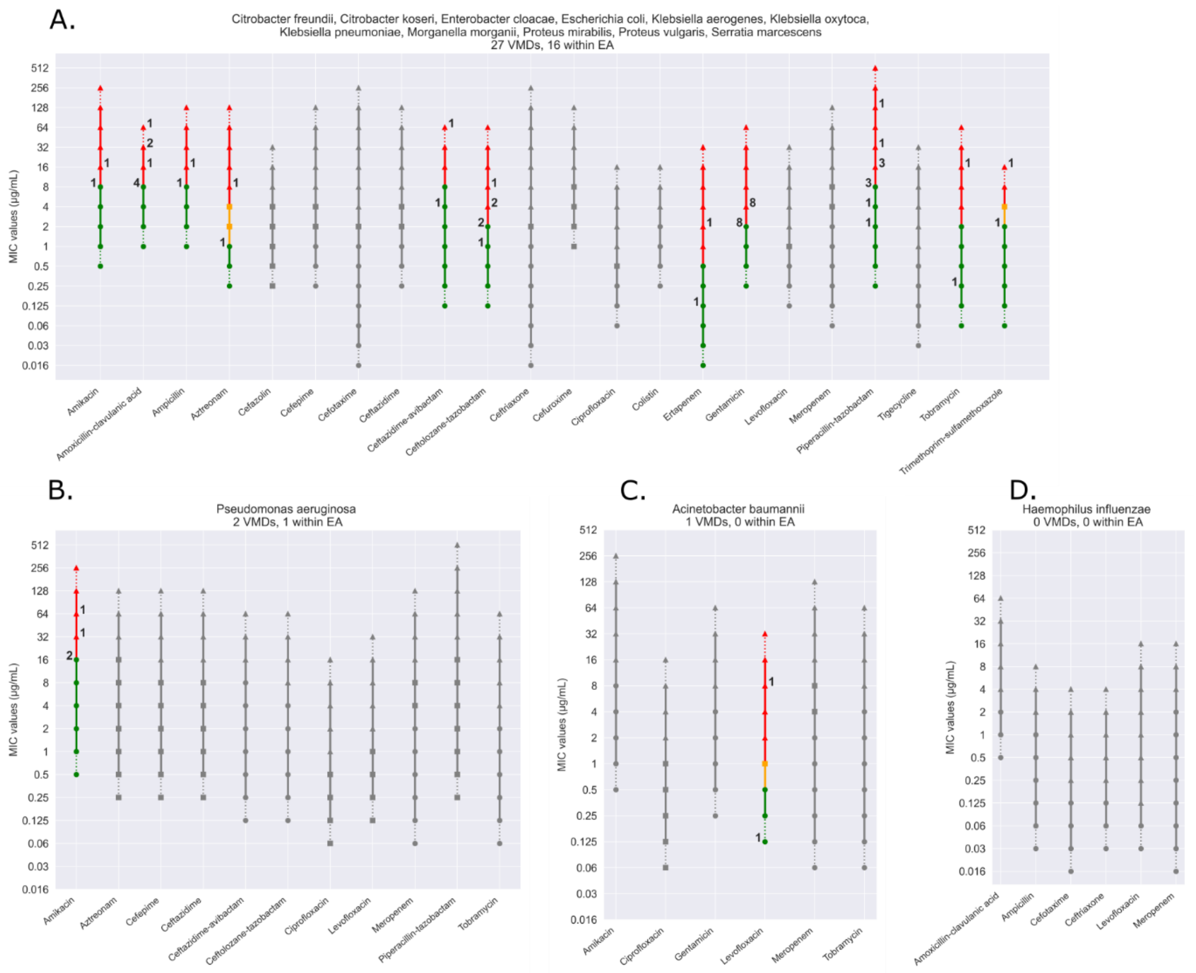

Supplementary Figure 3.

Very major discrepancy results from analysis using ASTar BC G- Kit software version 1.5 and interpreted according to discrepancy calculation strategy 1 and after discrepancy resolution. Where reference MIC ranges were broader than those of ASTar, they were truncated to ASTar ranges. In some instances reference MIC range was shorter than that of ASTar, primarily concerning CDC isolates. ASTar MIC results are presented to the left of the range line and corresponding reference MIC values are indicated to the right. Green indicates ‘S’, yellow ‘I’, and red ‘R’.

A. Enterobacterales

Very major discrepancies for Enterobacterales of more than one MIC value from reference MIC (non EA) were 3 amoxicillin-clavulanic acid, 1 aztreonam, 1 ceftazidime-avibactam, 1 ceftolozane-tazobactam, 1 ertapenem, 2 piperacillin-tazobactam, 1 tobramycin, and 1 trimethoprim-sulfamethoxazole.

B. *P. aeruginosa*

Very major discrepancies for *P. aeruginosa* of more than one MIC value from reference MIC (non EA) were 1 amikacin.

C. *A. baumannii*

Very major discrepancies for *A. baumannii* of more than one MIC value from reference MIC (non EA) were 1 levofloxacin.

D. *H. influenzae*

No very major discrepancies were registered for *H. influenzae.*

Supplementary Table 2. Presentation of performance characteristics data for screening agent cefoxitin. MIC >8 µg/mL is interpreted as a positive screening test for AmpC, using BMD for reference.

Antimicrobial agent	Positive agreement	Negative agreement
Cefoxitin	55/59 (93.2%)	245/266 (92.1%)

Positive agreement (proportion of correctly reported AmpC-positive isolates) = number of isolates tested positive by the ASTar System divided by number of isolates tested positive by reference method.

Negative agreement (Proportion of correctly reported AmpC-negative isolates) = number of isolates correctly tested negative by the ASTar System divided by number of isolates tested negative verified by reference method.

Supplementary Table 3. Listed cefoxitin results in non-agreement with the reference, including MIC values. The total number of datapoints for cefoxitin are 325, and number of datapoints in agreement are 300.

	Isolate	Agreement		MIC Value	
**Species**		**ASTar**	**Reference**	**ASTar**	**Reference**
Escherichia coli	HVI177	POS	NEG	= 16	= 4
Escherichia coli	HVI199	POS	NEG	= 16	= 4
Escherichia coli	HVI217	POS	NEG	= 16	= 4
Escherichia coli	HVI219	POS	NEG	= 16	= 4
Proteus mirabilis	HVI360	POS	NEG	= 16	= 4
Escherichia coli	ORE126	POS	NEG	= 16	= 4
Escherichia coli	ORE129	POS	NEG	= 32	= 4
Proteus mirabilis	ORE255	POS	NEG	= 16	= 4
Escherichia coli	P02046	POS	NEG	= 16	= 4
Escherichia coli	P02055	POS	NEG	= 16	= 4
Escherichia coli	HVI189	POS	NEG	= 16	= 8
Escherichia coli	HVI192	POS	NEG	= 16	= 8
Escherichia coli	HVI231	POS	NEG	= 16	= 8
Escherichia coli	HVI234	POS	NEG	= 16	= 8
Proteus mirabilis	HVI374	POS	NEG	= 16	= 8
Proteus mirabilis	HVI380	POS	NEG	= 16	= 8
Escherichia coli	ORE075	POS	NEG	= 16	= 8
Escherichia coli	ORE077	POS	NEG	= 16	= 8
Escherichia coli	ORE085	POS	NEG	= 16	= 8
Escherichia coli	ORE133	POS	NEG	= 16	= 8
Escherichia coli	QM565	POS	NEG	= 16	= 8
Klebsiella pneumoniae	HVI291	NEG	POS	= 8	= 16
Escherichia coli	P02018	NEG	POS	= 4	= 16
Escherichia coli	P02026	NEG	POS	= 8	= 16
Klebsiella pneumoniae	P02067	NEG	POS	= 8	= 16

Supplementary Table 4. Reproducibility of ASTar BC G- Kit per antimicrobial, pooled across instruments.

Antimicrobial agent	Results that fall within 1 dilution (+/-1) of the mode result divided by total number of results	
	Best-case[a] totals (%)	Worst-case[b] totals (%)
**Ampicillin**	71/71 (100%)	71/71 (100%)
**Amoxicillin-clavulanic acid**	105/105 (100%)	105/105 (100%)
**Piperacillin-tazobactam**	155/155 (100%)	155/155 (100%)
**Cefazolin**	53/53 (100%)	53/53 (100%)
**Cefepime**	68/68 (100%)	68/68 (100%)
**Cefotaxime**	156/156 (100%)	156/156 (100%)
**Cefoxitin**	70/70 (100%)	70/70 (100%)
**Ceftazidime**	104/104 (100%)	92/104 (88.5%)
**Ceftazidime-avibactam**	138/138 (100%)	138/138 (100%)
**Ceftolozane-tazobactam**	156/156 (100%)	156/156 (100%)
**Ceftriaxone**	155/156 (99.4%)	154/156 (98.7%)
**Cefuroxime**	70/70 (100%)	70/70 (100%)
**Ertapenem**	103/104 (99.0%)	93/104 (89.4%)
**Meropenem**	78/87 (89.7%)*	78/87 (89.7%)
**Aztreonam**	52/52 (100%)	52/52 (100%)
**Ciprofloxacin**	87/87 (100%)	86/87 (98.9%)
**Levofloxacin**	122/122 (100%)	122/122 (100%)
**Amikacin**	156/156 (100%)	148/156 (94.9%)
**Gentamicin**	139/139 (100%)	137/139 (98.6%)
**Tobramycin**	156/156 (100%)	156/156 (100%)
**Tigecycline**	52/53 (98.1%)	52/53 (98.1%)
**Colistin**	70/70 (100%)	70/70 (100%)
**Trimethoprim-sulfamethoxazole**	88/88 (100%)	73/88 (83.0%)

a) Best case calculation for reproducibility assuming any off-scale results are within one dilution from the adjacent on-scale result.

b) Worst case calculation for reproducibility assuming any off-scale results are more than one dilution from the

adjacent on-scale result.

* A reproducibility of 95.4% can be calculated if the reference MIC for one *E. cloacae* isolate is used instead of mode MIC.
